# Emergent Presentation of an Adult with Undiagnosed Coarctation of the Aorta

**DOI:** 10.1155/2018/5756983

**Published:** 2018-02-19

**Authors:** Shannon Armistead, David Mullins, Stephen Sherick, John Ashurst

**Affiliations:** ^1^West Virginia School of Osteopathic Medicine, 400 N. Lee St., Lewisburg, WV 24901, USA; ^2^Department of Emergency Medicine, Duke LifePoint Conemaugh Memorial Medical Center, 1086 Franklin Street, Johnstown, PA 15905, USA; ^3^Department of Emergency Medicine, Kingman Regional Medical Center, 3269 Stockton Hill Road, Kingman, AZ 86409, USA

## Abstract

Coarctation of the aorta is typically thought to be a childhood disease. However, emergency physicians must keep a broad differential diagnosis when faced with a young patient with signs and symptoms of acute congestive heart failure. The authors present a case of newly diagnosed coarctation of the aorta in a 26-year-old male who was first misdiagnosed with pneumonia.

## 1. Introduction

Coarctation of the aorta (CoA) is a narrowing of the aorta located frequently near the ligamentum arteriosum, a remnant of fetal circulation between the aorta and the pulmonary trunk distal to the left origin of the subclavian artery. The reported prevalence is approximately 4 in 10,000 livebirths and accounts for 4 to 6% of all congenital heart defects [1]. Aortic coarctation presenting during adult life most frequently represents cases of recoarctation, following previous transcatheter or surgical therapy, or missed cases of native coarctation [2]. The authors report a case of coarctation in a young adult undiagnosed until emergent presentation to the emergency department.

## 2. Report of the Case

A 26-year-old male presented to the emergency department complaining of shortness of breath with a cough and blood tinged sputum for the last week. He also reported associated left-sided chest pain that was described as a heaviness. The patient had presented to an urgicenter earlier during the week with similar symptoms and was diagnosed with pneumonia. He was given ciprofloxacin but noted worsening symptoms that prompted him to present to the emergency department.

His vital signs revealed a temperature of 36.7°C, blood pressure of 112/77, pulse of 96 beats per minute, respiratory rate of 20 breaths per minute, and pulse oximetry of 96% on room air. A vascular exam revealed the left radial pulse to be slightly weaker than the right radial pulse. Discovery of varied pulse strength led to bilateral upper extremity blood pressure being recorded as 141/77 on the right and 114/78 on the left. Mild bilateral rales were noted on pulmonary examination. The remainder of the examination was within normal limits.

An electrocardiogram displayed normal sinus rhythm with left ventricular hypertrophy. Laboratory testing showed unremarkable complete blood cell count and comprehensive metabolic panel. The patient's brain natriuretic peptide (BNP) revealed significant elevation of 1692 reported by the lab. Computed tomography (CT) of the chest revealed a 7 cm ascending aortic aneurysm with coarctation of the aorta, distal to the aortic arch at the level of the subclavian artery.

While in the emergency department, the patient was given a single dose of furosemide and was taken to cardiothoracic surgery for further evaluation in preparation for intervention. After review of the CT scan and discussion with cardiology, the patient was transferred to a tertiary referral center for surgical intervention.

## 3. Discussion

Coarctation of the aorta in adults accounts for 0.2% of all hypertension cases in adults [3]. Traditionally, adults with coarctation are asymptomatic until an insidious injury occurs and will typically present with acute heart failure, spinal complications, hypertensive crisis, or aortic complications [4].

A classic physical exam finding that can lead to the diagnosis of CoA is systolic blood pressure that is higher in the right arm compared to the right leg, while diastolic blood pressure is similar. When a coarctation is proximal to the left subclavian artery, systolic blood pressure is higher in the right arm than in the left arm [3]. This presentation is less common but, as in the case of our patient, is indicative of possible lesion location that an astute provider may correlate to diagnostic imaging.

When confirming a suspected CoA, all patients should have a chest radiograph to assess for rib notching and potential cardiomegaly. Echocardiography is essential for evaluation of structure and function of the left ventricle, as well as the aortic and mitral valves. CT or magnetic resonance imaging is now used to create three-dimensional images and to define and assess for collateral blood flow. Cardiac catheterization was once frequently used for the diagnosis of CoA but is now reserved for therapeutic interventions [5].

Management of previously undiagnosed CoA adults varies based primarily on the stability of the patient and severity of the lesion. The American College of Cardiology and the American Heart Association recommended intervention in adults with CoA in the following settings: when a peak-to-peak coarctation gradient ≥ 20 mg or peak-to-peak coarctation gradient < 20 mg with imaging evidence of significant coarctation and radiographic evidence of collateral flow [6]. When intervention is required in adults, stenting is the preferred approach at many centers [5].

## 4. Conclusion

Coarctation of the aorta, considered among most care providers as a pediatric issue, can present at any age. In adult patients, there can be little evidence of the pathology until the need for emergent care. Though this disease process is seen infrequently at best, it is important for clinicians to use the physical exam skills learned early in their education.

## References

[1] Hoffman J. I. E., Kaplan S. (2002). The incidence of congenital heart disease. *Journal of the American College of Cardiology*.

[2] Jurcut R., Daraban A. M., Lorber A. (2011). Coarctation of the aorta in adults: what is the best treatment? Case report and literature review.. *Journal of Medicine and Life*.

[3] Prisant L. M., Mawulawde K., Kapoor D., Joe C. (2004). Coarctation of the aorta: a secondary cause of hypertension.. *Journal of clinical hypertension (Greenwich, Conn.)*.

[4] Hazuková R., Cermakova E., Pleskot M., Hazuková R. (2016). Frequency of emergencies in adults due to unrecognized coarctation of the aorta. *Anatolian Journal of Cardiology/Anadolu Kardiyoloji Dergisi*.

[5] Torok R. D. (2015). Coarctation of the aorta: Management from infancy to adulthood. *World Journal of Cardiology*.

[6] Warnes C. A., Williams R. G., Bashore T. M. (2008). ACC/AHA 2008 guidelines for the management of adults with congenital heart disease: a report of the American College of Cardiology/American Heart Association Task Force on Practice Guidelines. *Circulation*.

